# 3-Cyano-*N*-(2-hy­droxy­benz­yl)anilinium nitrate

**DOI:** 10.1107/S1600536810054231

**Published:** 2011-01-08

**Authors:** Jing Dai, Xin-Yuan Chen

**Affiliations:** a Ordered Matter Science Research Center, College of Chemistry and Chemical Engineering, Southeast University, Nanjing 210096, People’s Republic of China

## Abstract

In the crystal structure of the title compound, C_14_H_13_N_2_O^+^·NO_3_^−^, N—H⋯O and O—H⋯O hydrogen bonds link cations and anions into a two-dimensional network parallel to (100). The dihedral angle between the rings is 9.48 (2)°.

## Related literature

For the properties and structures of related compounds, see: Fu *et al.* (2007 ▶, 2008 ▶, 2009 ▶); Fu & Xiong (2008 ▶).
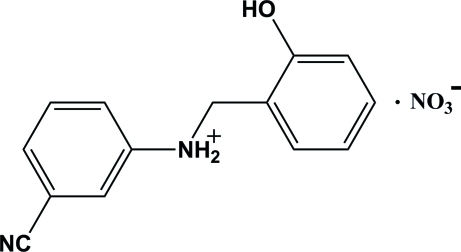


## Experimental

### 

#### Crystal data

C_14_H_13_N_2_O^+^·NO_3_^−^
*M* *_r_* = 287.27
Monoclinic, 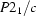

*a* = 12.060 (2) Å
*b* = 13.632 (3) Å
*c* = 8.8679 (18) Å
β = 93.71 (3)°
*V* = 1454.9 (5) Å^3^
*Z* = 4
Mo *K*α radiation
μ = 0.10 mm^−1^
*T* = 298 K
0.10 × 0.03 × 0.03 mm

#### Data collection

Rigaku Mercury2 diffractometer
Absorption correction: multi-scan (*CrystalClear*; Rigaku, 2005 ▶) *T*_min_ = 0.910, *T*_max_ = 1.000
14660 measured reflections
2857 independent reflections
1931 reflections with *I* > 2σ(*I*)
*R*_int_ = 0.053

#### Refinement

*R*[*F* ^2^ > 2σ(*F* ^2^)] = 0.066
*wR*(*F* ^2^) = 0.187
*S* = 1.06
2857 reflections
191 parameters
H-atom parameters constrained
Δρ_max_ = 0.44 e Å^−3^
Δρ_min_ = −0.17 e Å^−3^

### 

Data collection: *CrystalClear* (Rigaku, 2005 ▶); cell refinement: *CrystalClear*; data reduction: *CrystalClear*; program(s) used to solve structure: *SHELXS97* (Sheldrick, 2008 ▶); program(s) used to refine structure: *SHELXL97* (Sheldrick, 2008 ▶); molecular graphics: *ORTEPIII* (Burnett & Johnson, 1996 ▶), *ORTEP-3 for Windows* (Farrugia, 1997 ▶) and *XP* in *SHELXTL* (Sheldrick, 2008 ▶); software used to prepare material for publication: *SHELXTL*.

## Supplementary Material

Crystal structure: contains datablocks I, global. DOI: 10.1107/S1600536810054231/dn2642sup1.cif

Structure factors: contains datablocks I. DOI: 10.1107/S1600536810054231/dn2642Isup2.hkl

Additional supplementary materials:  crystallographic information; 3D view; checkCIF report

## Figures and Tables

**Table 1:** Hydrogen-bond geometry (Å, °)

*D*—H⋯*A*	*D*—H	H⋯*A*	*D*⋯*A*	*D*—H⋯*A*
O1—H1⋯O2^i^	0.82	1.93	2.753 (3)	177
N1—H1*A*⋯O4	0.90	2.10	2.936 (3)	155
N1—H1*A*⋯O3	0.90	2.49	3.209 (3)	137
N1—H1*B*⋯O2^ii^	0.90	1.97	2.824 (3)	158

Symmetry codes: (i) 
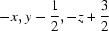
; (ii) 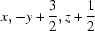.

**Fig. 1.**
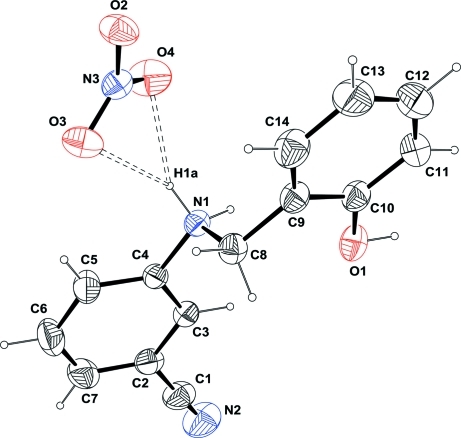
A view of the title compound with the atomic numbering scheme. Displacement ellipsoids are drawn at the 30% probability level. H atoms are represented as small spheres of arbitrary radii. Bifurcated hydrogen bonds are shown as dashed lines.

**Fig. 2.**
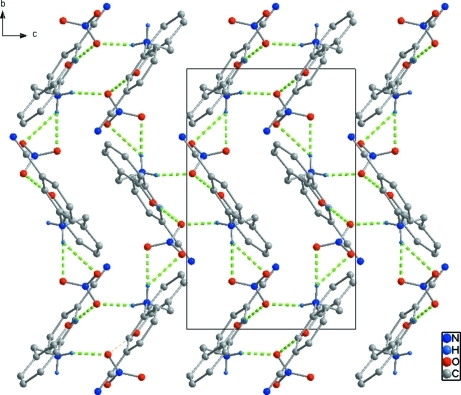
The crystal packing of the title compound, showing the two-dimensional network. H atoms not involved in hydrogen bonding (dashed line) have been omitted for clarity.

## supplementary crystallographic information

### Comment

Salts of amine attracted more attention as phase transition dielectric materials for its application in memory storage (Fu *et al.* 2007; Fu & Xiong 2008; Fu *et al.* 2008; Fu *et al.* 2009). With the purpose of obtaining phase transition crystals of 3-(2-hydroxybenzylamino)benzonitrile salts, its interaction with various acids has been studied and we have elaborated a series of new materials with this organic molecule. In this study, we describe the crystal structure of the title compound, 3-cyano-N-(2-hydroxybenzyl)anilinium nitrate.

The dielectric constant of title compound as a function of temperature indicates that the permittivity is basically temperature-independent, suggesting that this compound should be not a real ferroelectrics or there may be no distinct phase transition occurred within the measured temperature range. Similarly, below the melting point (438 K) of the compound, the dielectric constant as a function of temperature also goes smoothly, and there is no dielectric anomaly observed (dielectric constant ranging from 6.1 to 7.3).

The asymmetric unit is composed of one NO_3_^-^ anion and one C_14_H_13_ON_2_^+^ cation (Fig.1). The amine N atom was protonated, thus indicating a positive charge. And the NO_3_^-^ anion was showing a negative charge to make the charge balance. The geometric parameters of the title compound are in the normal range.

In the crystal structure, all the H atoms of N atom and the hydroxyl are involved in hydrogen bonds. One of the H atom of the NH_2_ group is giving a bifurcated N—H···O hydrogen bonds with O3 and O4 atoms of NO_3_^-^, respectively. The another H atom of the NH_2_ group and the H atoms of hydroxyl are involved in N—H···O and O—H···O hydrogen bonds with the O2 atom of the NO_3_^-^. These hydrogen bonds link the ionic units into a two-dimentional network parallel to the (1 0 0) plane. (Table 1 and Fig.2).

### Experimental

The commercial 3-(2-hydroxybenzylamino)benzonitrile (3 mmol) was dissolved in water/HNO_3_ (50:1 *v*/*v*) solution. The solvent was slowly evaporated in air affording colourless block-shaped crystals of the title compound suitable for X-ray analysis.

### Refinement

All H atoms attached to C, N and O atoms were fixed geometrically and treated as riding with C–H = 0.93 Å (Caromatic) or 0.97 Å (Cmethylene), N–H = 0.90 Å and O–H = 0.82 Å with U_iso_(H) = 1.2U_eq_(C,N) or U_iso_(H) = 1.5U_eq_(O).

### Crystal data

**Table:**

C_14_H_13_N_2_O^+^·NO_3_^−^	*F*(000) = 600
*M**_r_* = 287.27	*D*_x_ = 1.312 Mg m^−^^3^
Monoclinic, *P*2_1_/*c*	Mo *K*α radiation, λ = 0.71073 Å
Hall symbol: -P 2ybc	Cell parameters from 3326 reflections
*a* = 12.060 (2) Å	θ = 3.0–27.5°
*b* = 13.632 (3) Å	µ = 0.10 mm^−^^1^
*c* = 8.8679 (18) Å	*T* = 298 K
β = 93.71 (3)°	Block, colorless
*V* = 1454.9 (5) Å^3^	0.10 × 0.03 × 0.03 mm
*Z* = 4	

### Data collection

**Table:**

Rigaku Mercury2 diffractometer	2857 independent reflections
Radiation source: fine-focus sealed tube	1931 reflections with *I* > 2σ(*I*)
graphite	*R*_int_ = 0.053
Detector resolution: 13.6612 pixels mm^-1^	θ_max_ = 26.0°, θ_min_ = 3.0°
CCD profile fitting scans	*h* = −14→14
Absorption correction: multi-scan (*CrystalClear*; Rigaku, 2005)	*k* = 0→16
*T*_min_ = 0.910, *T*_max_ = 1.000	*l* = 0→10
14660 measured reflections	

### Refinement

**Table:**

Refinement on *F*^2^	Primary atom site location: structure-invariant direct methods
Least-squares matrix: full	Secondary atom site location: difference Fourier map
*R*[*F*^2^ > 2σ(*F*^2^)] = 0.066	Hydrogen site location: inferred from neighbouring sites
*wR*(*F*^2^) = 0.187	H-atom parameters constrained
*S* = 1.06	*w* = 1/[σ^2^(*F*_o_^2^) + (0.0773*P*)^2^ + 0.7478*P*] where *P* = (*F*_o_^2^ + 2*F*_c_^2^)/3
2857 reflections	(Δ/σ)_max_ < 0.001
191 parameters	Δρ_max_ = 0.44 e Å^−^^3^
0 restraints	Δρ_min_ = −0.17 e Å^−^^3^

### Special details

**Table:**

Geometry. All esds (except the esd in the dihedral angle between two l.s. planes) are estimated using the full covariance matrix. The cell esds are taken into account individually in the estimation of esds in distances, angles and torsion angles; correlations between esds in cell parameters are only used when they are defined by crystal symmetry. An approximate (isotropic) treatment of cell esds is used for estimating esds involving l.s. planes.
Refinement. Refinement of F^2^ against ALL reflections. The weighted R-factor wR and goodness of fit S are based on F^2^, conventional R-factors R are based on F, with F set to zero for negative F^2^. The threshold expression of F^2^ > 2sigma(F^2^) is used only for calculating R-factors(gt) etc. and is not relevant to the choice of reflections for refinement. R-factors based on F^2^ are statistically about twice as large as those based on F, and R- factors based on ALL data will be even larger.

### Fractional atomic coordinates and isotropic or equivalent isotropic displacement parameters (Å^2^)

**Table:**

	*x*	*y*	*z*	*U*_iso_*/*U*_eq_	
O1	0.01326 (17)	0.48563 (16)	0.7983 (2)	0.0641 (6)	
H1	−0.0322	0.4598	0.8506	0.096*	
N1	0.21801 (17)	0.60209 (15)	0.7364 (2)	0.0441 (5)	
H1A	0.2317	0.6669	0.7312	0.053*	
H1B	0.1804	0.5914	0.8192	0.053*	
N2	0.4445 (3)	0.2610 (2)	1.0265 (5)	0.1039 (12)	
C1	0.4388 (3)	0.3282 (3)	0.9493 (5)	0.0740 (10)	
C2	0.4304 (2)	0.4142 (2)	0.8546 (3)	0.0561 (7)	
C3	0.3305 (2)	0.46590 (19)	0.8403 (3)	0.0490 (7)	
H3A	0.2691	0.4443	0.8893	0.059*	
C4	0.3240 (2)	0.54951 (19)	0.7526 (3)	0.0433 (6)	
C5	0.4133 (2)	0.5825 (2)	0.6785 (4)	0.0625 (8)	
H5A	0.4077	0.6390	0.6197	0.075*	
C6	0.5124 (3)	0.5300 (3)	0.6929 (4)	0.0796 (10)	
H6A	0.5736	0.5520	0.6439	0.096*	
C7	0.5207 (3)	0.4464 (3)	0.7782 (4)	0.0717 (9)	
H7A	0.5868	0.4110	0.7850	0.086*	
C8	0.1457 (2)	0.5717 (2)	0.5977 (3)	0.0502 (7)	
H8A	0.1749	0.6002	0.5083	0.060*	
H8B	0.1474	0.5009	0.5874	0.060*	
C9	0.0277 (2)	0.6051 (2)	0.6099 (3)	0.0533 (7)	
C10	−0.0376 (2)	0.5602 (2)	0.7136 (3)	0.0544 (7)	
C11	−0.1472 (3)	0.5889 (2)	0.7271 (4)	0.0681 (9)	
H11A	−0.1907	0.5586	0.7964	0.082*	
C12	−0.1896 (3)	0.6633 (3)	0.6353 (5)	0.0850 (11)	
H12A	−0.2629	0.6829	0.6435	0.102*	
C13	−0.1275 (3)	0.7091 (3)	0.5326 (5)	0.0876 (12)	
H13A	−0.1578	0.7598	0.4731	0.105*	
C14	−0.0188 (3)	0.6792 (2)	0.5179 (4)	0.0724 (9)	
H14A	0.0233	0.7088	0.4464	0.087*	
O2	0.14630 (18)	0.90283 (15)	0.5332 (2)	0.0654 (6)	
O3	0.2686 (2)	0.78798 (16)	0.5296 (3)	0.0731 (7)	
O4	0.1883 (2)	0.81584 (17)	0.7324 (3)	0.0849 (8)	
N3	0.20210 (19)	0.83479 (17)	0.5999 (3)	0.0507 (6)	

### Atomic displacement parameters (Å^2^)

**Table:**

	*U*^11^	*U*^22^	*U*^33^	*U*^12^	*U*^13^	*U*^23^
O1	0.0621 (13)	0.0672 (13)	0.0624 (13)	−0.0158 (11)	−0.0006 (10)	0.0150 (10)
N1	0.0452 (12)	0.0400 (11)	0.0470 (12)	−0.0023 (9)	0.0019 (9)	0.0014 (9)
N2	0.103 (3)	0.068 (2)	0.137 (3)	0.0141 (18)	−0.023 (2)	0.021 (2)
C1	0.064 (2)	0.0563 (19)	0.099 (3)	0.0114 (16)	−0.0158 (18)	−0.0036 (19)
C2	0.0498 (16)	0.0494 (16)	0.0671 (19)	0.0044 (13)	−0.0115 (14)	−0.0084 (14)
C3	0.0471 (15)	0.0449 (15)	0.0549 (16)	−0.0032 (12)	0.0026 (12)	−0.0032 (12)
C4	0.0387 (14)	0.0440 (14)	0.0467 (14)	−0.0022 (11)	−0.0011 (11)	−0.0079 (11)
C5	0.0516 (17)	0.066 (2)	0.071 (2)	−0.0077 (15)	0.0078 (14)	0.0057 (15)
C6	0.0445 (18)	0.099 (3)	0.097 (3)	−0.0055 (18)	0.0136 (17)	−0.001 (2)
C7	0.0461 (18)	0.077 (2)	0.090 (2)	0.0110 (16)	−0.0057 (16)	−0.012 (2)
C8	0.0500 (16)	0.0561 (16)	0.0441 (15)	−0.0054 (13)	0.0000 (12)	−0.0036 (12)
C9	0.0527 (16)	0.0524 (16)	0.0541 (16)	−0.0072 (13)	−0.0036 (13)	−0.0028 (13)
C10	0.0521 (16)	0.0531 (16)	0.0571 (17)	−0.0073 (13)	−0.0033 (13)	−0.0035 (14)
C11	0.0539 (19)	0.068 (2)	0.083 (2)	−0.0026 (16)	0.0103 (16)	−0.0027 (17)
C12	0.062 (2)	0.075 (2)	0.118 (3)	0.0126 (19)	−0.002 (2)	0.006 (2)
C13	0.073 (2)	0.067 (2)	0.120 (3)	0.0169 (19)	−0.020 (2)	0.014 (2)
C14	0.071 (2)	0.067 (2)	0.078 (2)	−0.0063 (17)	−0.0055 (17)	0.0138 (17)
O2	0.0761 (14)	0.0616 (13)	0.0597 (12)	0.0242 (11)	0.0124 (10)	0.0147 (10)
O3	0.0754 (15)	0.0667 (14)	0.0767 (15)	0.0236 (12)	0.0015 (12)	−0.0026 (12)
O4	0.129 (2)	0.0768 (16)	0.0495 (13)	0.0135 (15)	0.0104 (13)	0.0155 (11)
N3	0.0569 (14)	0.0443 (13)	0.0501 (14)	−0.0026 (11)	−0.0032 (11)	0.0023 (11)

### Geometric parameters (Å, °)

**Table:**

O1—C10	1.383 (3)	C7—H7A	0.9300
O1—H1	0.8200	C8—C9	1.504 (4)
N1—C4	1.465 (3)	C8—H8A	0.9700
N1—C8	1.518 (3)	C8—H8B	0.9700
N1—H1A	0.9000	C9—C10	1.391 (4)
N1—H1B	0.9000	C9—C14	1.393 (4)
N2—C1	1.143 (5)	C10—C11	1.391 (4)
C1—C2	1.442 (5)	C11—C12	1.378 (5)
C2—C7	1.390 (5)	C11—H11A	0.9300
C2—C3	1.395 (4)	C12—C13	1.368 (6)
C3—C4	1.379 (4)	C12—H12A	0.9300
C3—H3A	0.9300	C13—C14	1.387 (5)
C4—C5	1.373 (4)	C13—H13A	0.9300
C5—C6	1.391 (5)	C14—H14A	0.9300
C5—H5A	0.9300	O2—N3	1.269 (3)
C6—C7	1.369 (5)	O3—N3	1.226 (3)
C6—H6A	0.9300	O4—N3	1.225 (3)
			
C10—O1—H1	109.5	C9—C8—H8A	109.5
C4—N1—C8	113.48 (19)	N1—C8—H8A	109.5
C4—N1—H1A	108.9	C9—C8—H8B	109.5
C8—N1—H1A	108.9	N1—C8—H8B	109.5
C4—N1—H1B	108.9	H8A—C8—H8B	108.1
C8—N1—H1B	108.9	C10—C9—C14	118.8 (3)
H1A—N1—H1B	107.7	C10—C9—C8	119.5 (3)
N2—C1—C2	178.7 (4)	C14—C9—C8	121.6 (3)
C7—C2—C3	119.7 (3)	O1—C10—C11	123.4 (3)
C7—C2—C1	120.9 (3)	O1—C10—C9	115.6 (3)
C3—C2—C1	119.4 (3)	C11—C10—C9	121.0 (3)
C4—C3—C2	119.1 (3)	C12—C11—C10	118.3 (3)
C4—C3—H3A	120.5	C12—C11—H11A	120.8
C2—C3—H3A	120.5	C10—C11—H11A	120.8
C5—C4—C3	121.5 (3)	C13—C12—C11	122.1 (3)
C5—C4—N1	120.0 (2)	C13—C12—H12A	119.0
C3—C4—N1	118.5 (2)	C11—C12—H12A	119.0
C4—C5—C6	118.9 (3)	C12—C13—C14	119.3 (3)
C4—C5—H5A	120.6	C12—C13—H13A	120.3
C6—C5—H5A	120.6	C14—C13—H13A	120.3
C7—C6—C5	120.7 (3)	C13—C14—C9	120.4 (3)
C7—C6—H6A	119.6	C13—C14—H14A	119.8
C5—C6—H6A	119.6	C9—C14—H14A	119.8
C6—C7—C2	120.1 (3)	O4—N3—O3	120.8 (2)
C6—C7—H7A	120.0	O4—N3—O2	120.0 (2)
C2—C7—H7A	120.0	O3—N3—O2	119.2 (2)
C9—C8—N1	110.9 (2)		

### Hydrogen-bond geometry (Å, °)

**Table:**

*D*—H···*A*	*D*—H	H···*A*	*D*···*A*	*D*—H···*A*
O1—H1···O2^i^	0.82	1.93	2.753 (3)	177
N1—H1A···O4	0.90	2.10	2.936 (3)	155
N1—H1A···O3	0.90	2.49	3.209 (3)	137
N1—H1B···O2^ii^	0.90	1.97	2.824 (3)	158

Symmetry codes: (i) −*x*, *y*−1/2, −*z*+3/2; (ii) *x*, −*y*+3/2, *z*+1/2.
